# Supplementary material for: Real-time imaging of sulfhydryl single-stranded DNA aggregation
Source: Commun Chem. 2023 May 2;6:86. doi: 10.1038/s42004-023-00886-6 (PMC10154300; doi:10.1038/s42004-023-00886-6)
Supplement: Supplementary file 2 — Supplementary Information [file 42004_2023_886_MOESM2_ESM.pdf]

Supplementary Figure S1–S2

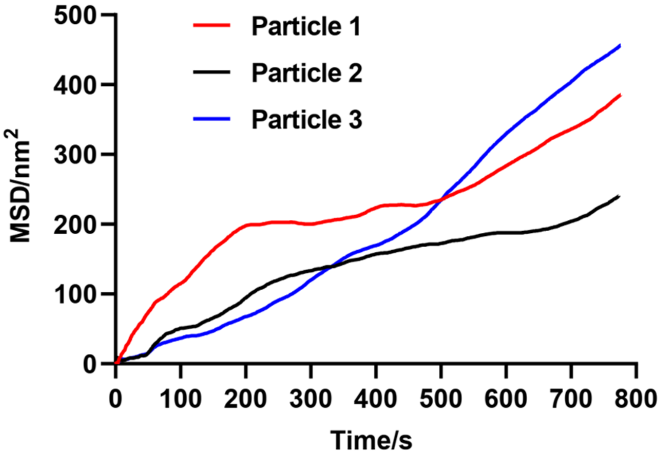

Supplementary Figure 1 | Mean square displacement (MSD) as a function of the elapsed time obtained from trajectories of moving nanoparticles of 5 μM SH-ssDNA macromolecule.

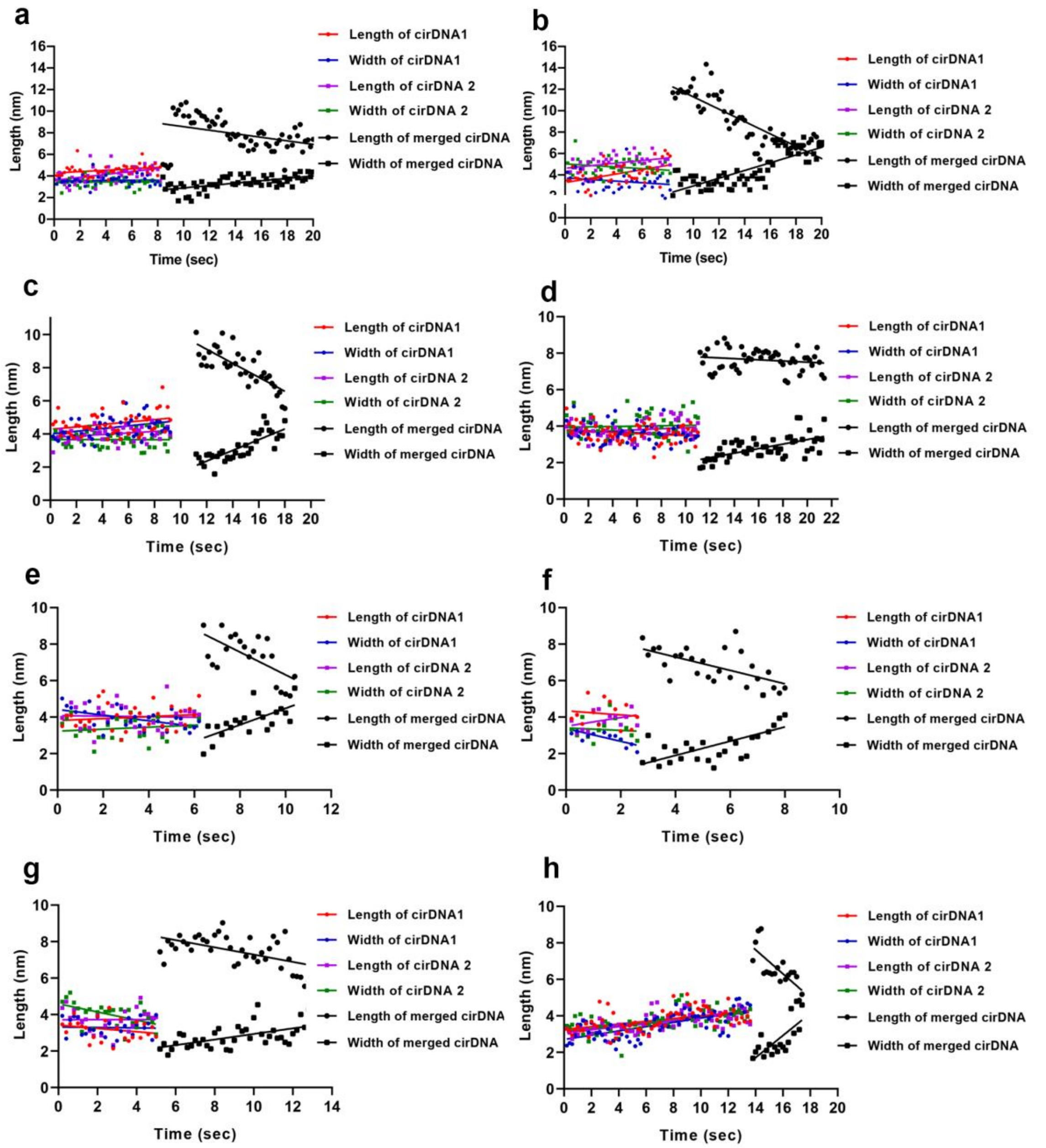

Supplementary Figure 2 | (a-f) Scatter plot showing the length and width of eight pairs of two smaller SS-cirDNA macromolecules as a function of time.
