# Supplementary material for: Real-time imaging of sulfhydryl single-stranded DNA aggregation
Source: Commun Chem. 2023 May 2;6:86. doi: 10.1038/s42004-023-00886-6 (PMC10154300; doi:10.1038/s42004-023-00886-6)
Supplement: Supplementary file 3 — Description of Additional Supplementary File [file 42004_2023_886_MOESM3_ESM.pdf]

Description of Additional Supplementary Files

File name: Supplementary Video 1

Description: In situ TEM observation of the ssDNA dynamic process in liquid (displayed at a 6× speed of the real-time process)

File name: Supplementary Video 2

Description: In situ TEM observation of SH-ssDNA self-assembly controlled by disulfide bonds in liquid (displayed at a 6× speed of the real-time process)

File name: Supplementary Video 3

Description: In situ TEM observation of the 0.5 µMSH-ssDNA dynamic process in liquid (displayed at a 6× speed of the real-time process)

File name: Supplementary Video 4

Description: In situ TEM observation of the 5 µMSH-ssDNA dynamic process in liquid (displayed at a 6× speed of the real-time process)

File name: Supplementary Video 5

Description: In situ TEM observation of one SS-cirDNA macromolecule merging with few SS-cirDNA macromolecules (displayed at a 6× speed of the real-time process)

File name: Supplementary Video 6

Description: In situ TEM observation of one SS-cirDNA macromolecule formation from two smaller SS-cirDNA macromolecule collisions (displayed at a6×speed of the real-time process)

File name: Supplementary Video 7

Description: In situ TEM observation of the structural transformation of SS-cirDNA in liquid under a higher magnification (displayed at a 6× speed of the real-time process)
